# Arthroscopic Management of Juxta‐Articular Proximal Tibial Chondroblastoma: A Case Report and Literature Review

**DOI:** 10.1111/os.14287

**Published:** 2024-11-07

**Authors:** Cong Xiao, Shaoyun Zhang, Zhixiang Gao, Lifu Wang, Yixin Dai, Jian Li

**Affiliations:** ^1^ Department of Orthopedics The Third Hospital of Mianyang, Sichuan Mental Health Center Mianyang People's Republic of China; ^2^ Sports Medicine Center, Department of Orthopedic Surgery Orthopedic Research Institute, West China Hospital, Sichuan University Chengdu People's Republic of China

**Keywords:** arthroscopy, bone substitute, chondroblastoma, proximal tibial

## Abstract

**Background:**

Chondroblastoma is a rare bone tumor that originates from the epiphysis, constitutes around 1% of all primary bone tumors and is recognized for its tendency to exhibit local invasiveness, as well as the possibility of metastasis and recurrence in nearby areas. Currently, the main surgical treatment for chondroblastoma is open surgery, involving excision of the lesion. There are relatively few reports on arthroscopic surgery for the treatment of chondroblastoma. However, open surgical curettage is associated with operation‐related trauma and potential for damage to the osteoepiphysis resulting in growth disturbances.

**Case Presentation:**

This case study presents the application of an arthroscopic technique in a 14‐year‐old male patient with chondroblastoma affecting the proximal tibia and tibial eminence. The procedure involved thorough removal of the lesion using direct visualization with the management of the cavity utilizing a substitute for autologous bone graft. After 1 year of follow‐up, the patient remains free from symptoms, exhibits normal knee functionality, and radiographic analysis reveals a good autologous bone graft fusion without any signs of recurrence.

**Conclusions:**

Based on the existing cases of arthroscopic treatment for chondroblastoma and the report of this case, arthroscopic treatment for chondroblastoma can be considered as a specific treatment option for certain patients. In some cases, this technique could be an effective alternative to open surgery.

## Graphical abstract

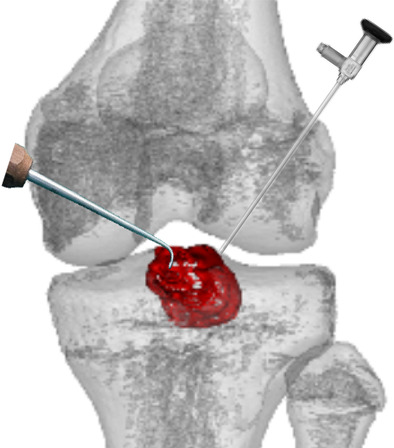

## 1 Background

The prevalence of chondroblastoma among primary bone tumors is estimated to be approximately 1%, predominantly affecting individuals aged between 10 and 30 years [1]. In a multi‐center retrospective study [2], it was reported that the mean age at the time of diagnosis was 18.02 ± 5.28 years. Regarding gender distribution, male patients were affected almost twice as frequently as female patients. Chondroblastoma is generally classified as an intermediate tumor category, characterized by its benign nature and rare metastasis, with a recurrence rate ranging from 5% to 40%. [2, 3, 4]. The first documented case of chondroblastoma dates back to 1942 [5]. Typically, chondroblastoma occurs in the epiphysis of a long bone, with the most common sites being the proximal tibia or femur, distal femur, and proximal humerus [2]. Though open surgical curettage with or without bone grafting is the mainstay of treatment, it is associated with a more pronounced operation‐related trauma [6]; or damage to the physis during unchecked curettage [7, 8]. The arthroscopic treatment offers distinct advantages in terms of enhanced visualization and minimal invasiveness when compared to open surgery, thereby facilitating early mobilization and yielding superior functional and cosmetic outcomes during the postoperative period.

According to our current knowledge, there has been only one reported case who underwent arthroscopic treatment for a proximal tibial chondroblastoma [9]. In this study, we present a case of arthroscopic management for a proximal tibial chondroblastoma, involving curettage under direct visualization followed by cavity treatment using an autologous bone graft substitute.

## 2 Case Presentation

**FIGURE 1:**
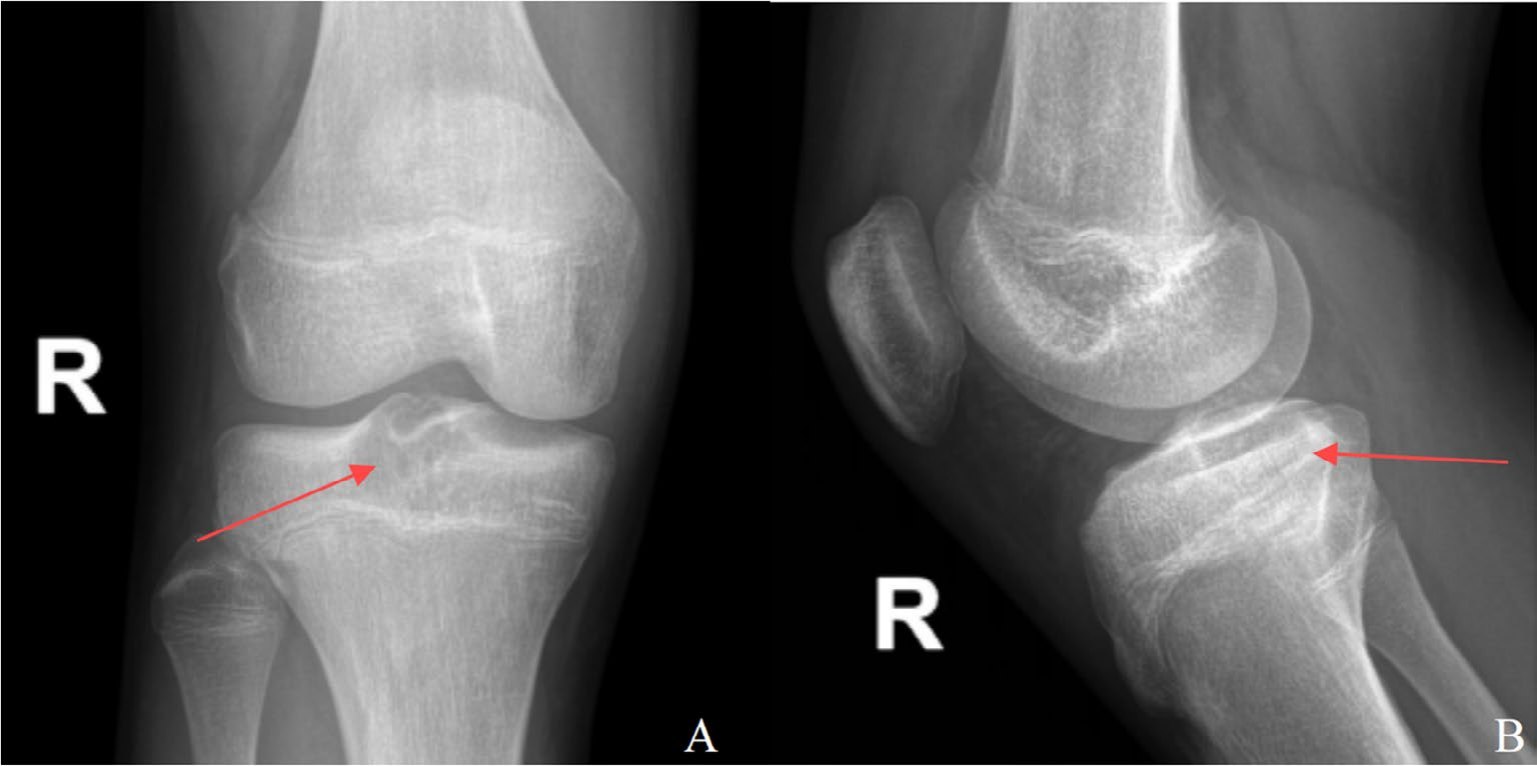
Radiographs of right (R) knee showing eccentric juxta‐articular osteolytic lesion in proximal tibial: Anteroposterior (A) and lateral (B) views.

**FIGURE 2:**
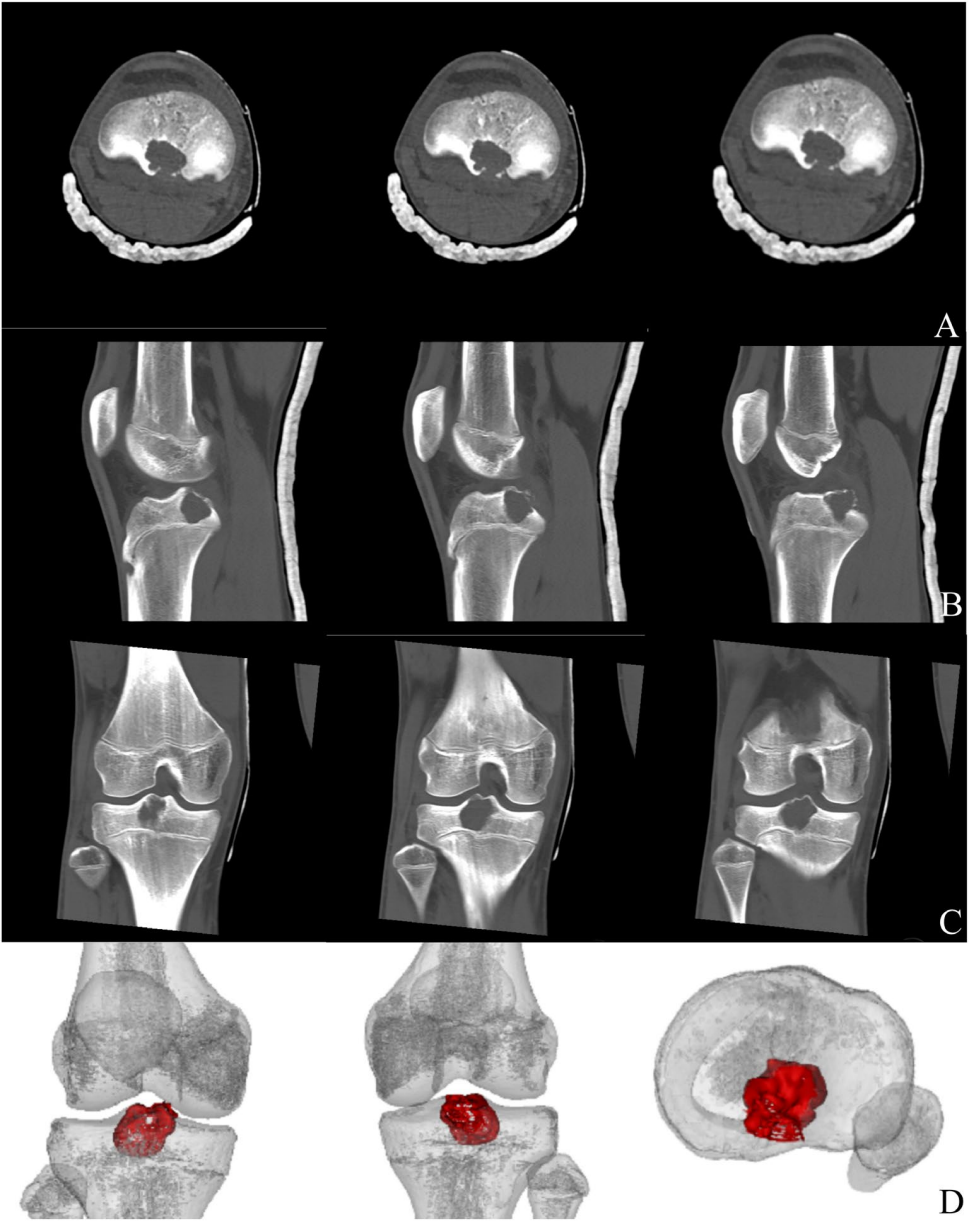
CT scans and three‐dimensional reconstruction showing osteolytic lesion in proximal tibial physis of right knee: axial view (A); sagittal view (B); coronal view (C); and three‐dimensional reconstruction of the lesion (D).

**FIGURE 3:**
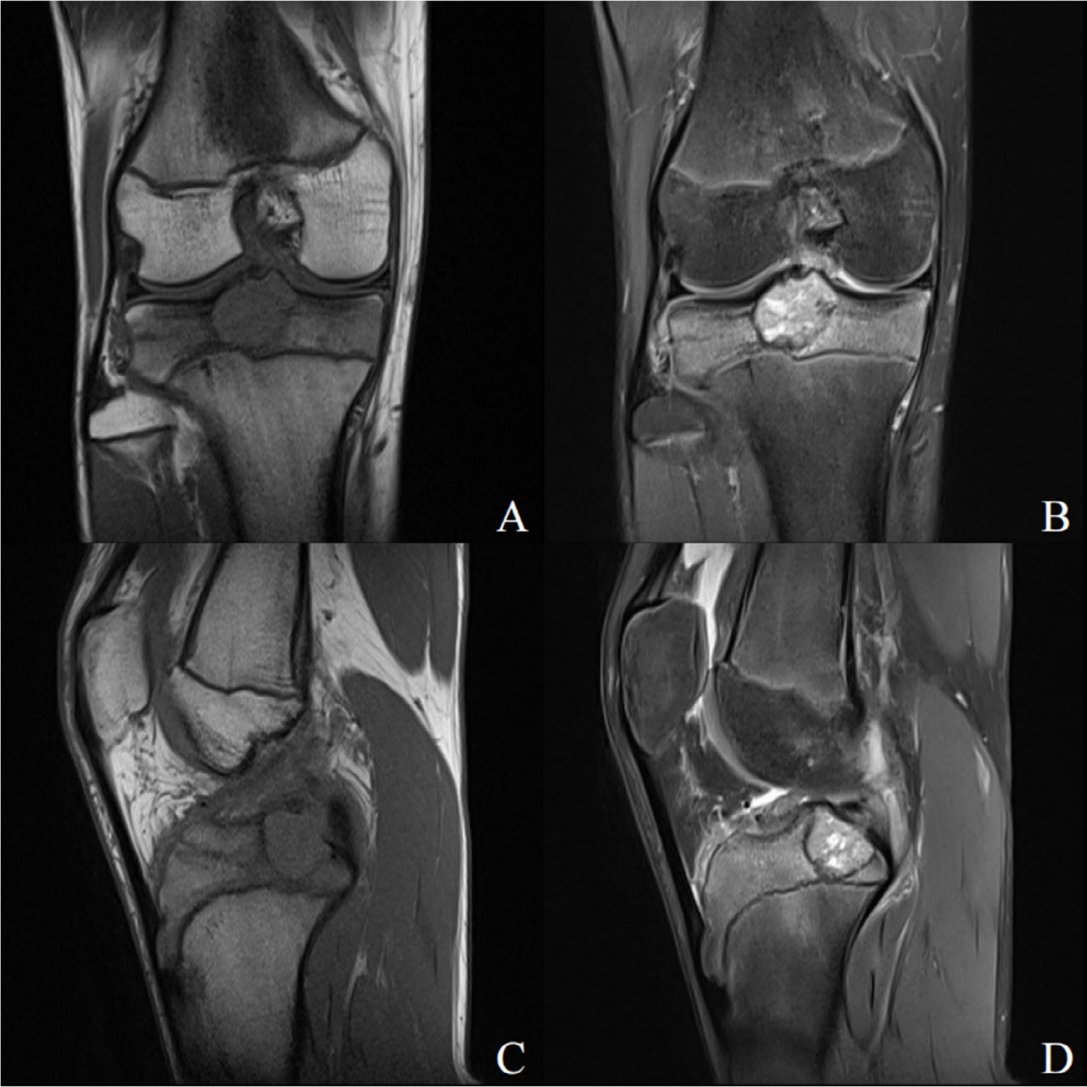
Magnetic resonance images of proximal tibial lesion in right knee: coronal T1‐weighted image (A), coronal T2‐weighted image view (B), sagittal T1‐weighted image (C), and sagittal T2‐weighted image (D). A well‐circumscribed septate lesion is seen, consisting of lobules with evidence of neighboring marrow edema.

**FIGURE A1:**
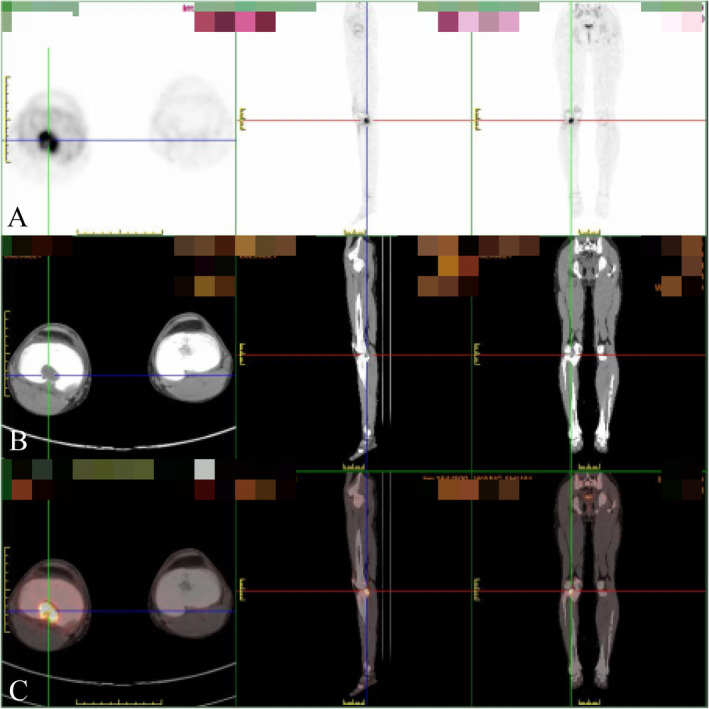
The ^18^F‐FDG PET/CT imaging of patient at our hospital, the SUVmax = 8.1.

A 14‐year‐old boy presented with pain in the right knee after running for 10 days. There was no history of any trauma, and the patient was in good health otherwise. The physical examination was unremarkable, except for the presence of pain upon flexion and extension of the right knee, as well as limited range of motion in the right knee joint with a maximum flexion of 90°. Radiographs revealed a well‐defined radiolucent lesion with sclerotic margins in the tibia intercondylar eminence of the right knee (Figure 1). Subsequent computed tomography (CT) scans and three‐dimensional film revealed an eccentric osteolytic lesion located posterior to the tibial intercondylar eminence, exhibiting a well‐defined lucency and marginal sclerosis (Figure 2). The magnetic resonance imaging (MRI) reveals a well‐defined septate lesion in the tibial eminence, comprising of lobules that exhibit hypointense on T1‐weighted imaging and hyperintense on T2‐weighted image. The presence of adjacent marrow edema is evident, while no involvement is detected in the tibial epiphyseal region (Figure 3). The patient underwent ^18^F‐FDG PET/CT imaging at our hospital, the SUVmax = 8.1, suggesting a primary bone tumor (Figure A1). Based on the evaluation of MRI, CT, and PET‐CT images, it is probable that this lesion represents a primary bone tumor with a high likelihood of being benign. Rather than the conventional arthrotomy, we chose an arthroscopic approach for decreased post‐operative pain that would facilitate early mobilization. The treatment strategy involves using arthroscopy to treat the cavity with an autologous bone graft substitute as the primary approach. If an intraoperative diagnosis indicates a malignant lesion, there will be a subsequent transition to arthroscopy‐guided biopsy.

**FIGURE 4:**
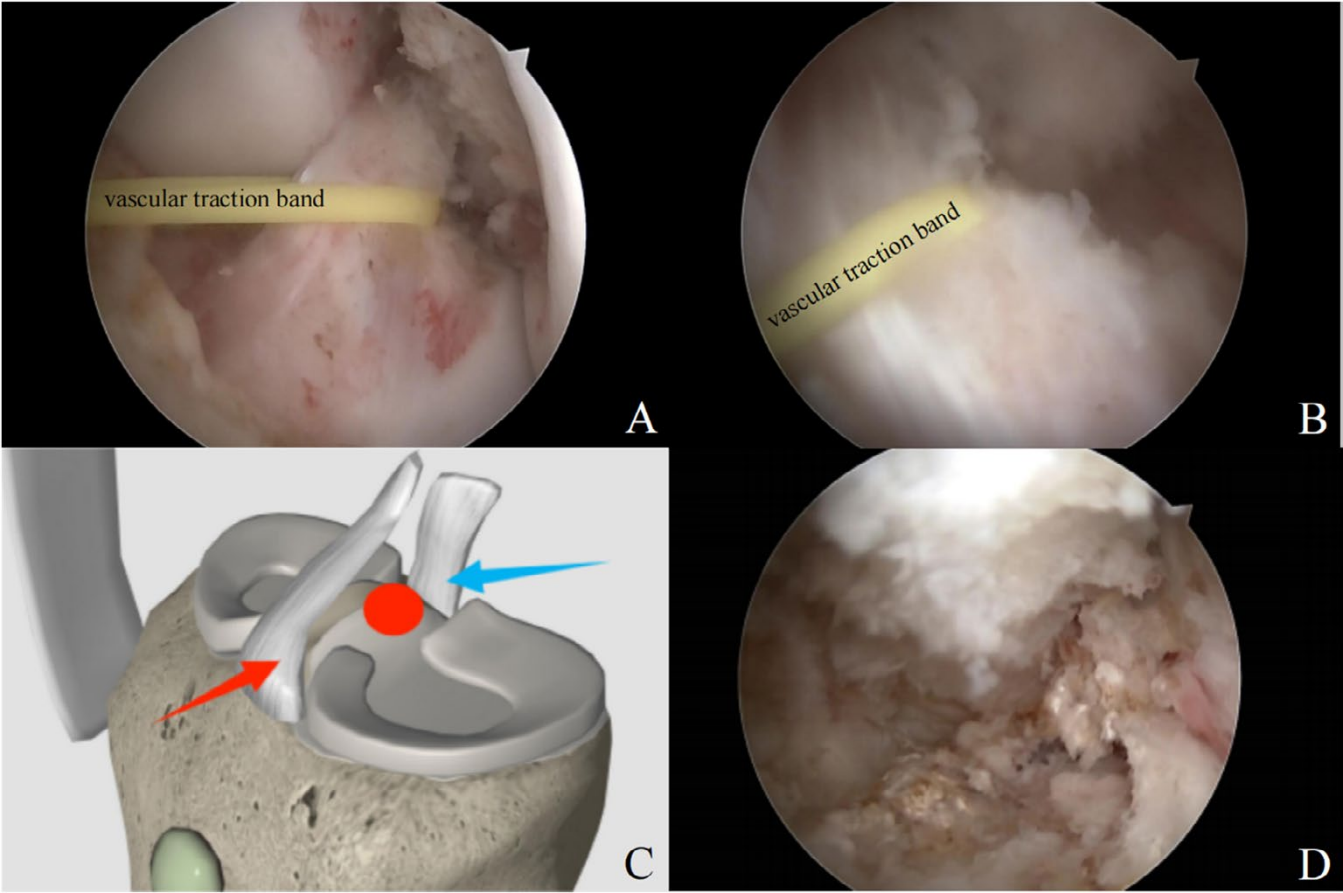
Arthroscopic procedures under direct vision: a vascular traction band is utilized to pull the anterior cruciate ligament (A, B); and the location of the lesion in knee (C), the red arrow represents the anterior cruciate ligament, the blue arrow represents the posterior cruciate ligament. The red circle indicates the location of the lesion; the lesion was exposed after soft tissues were cleaned (D).

**FIGURE 5:**
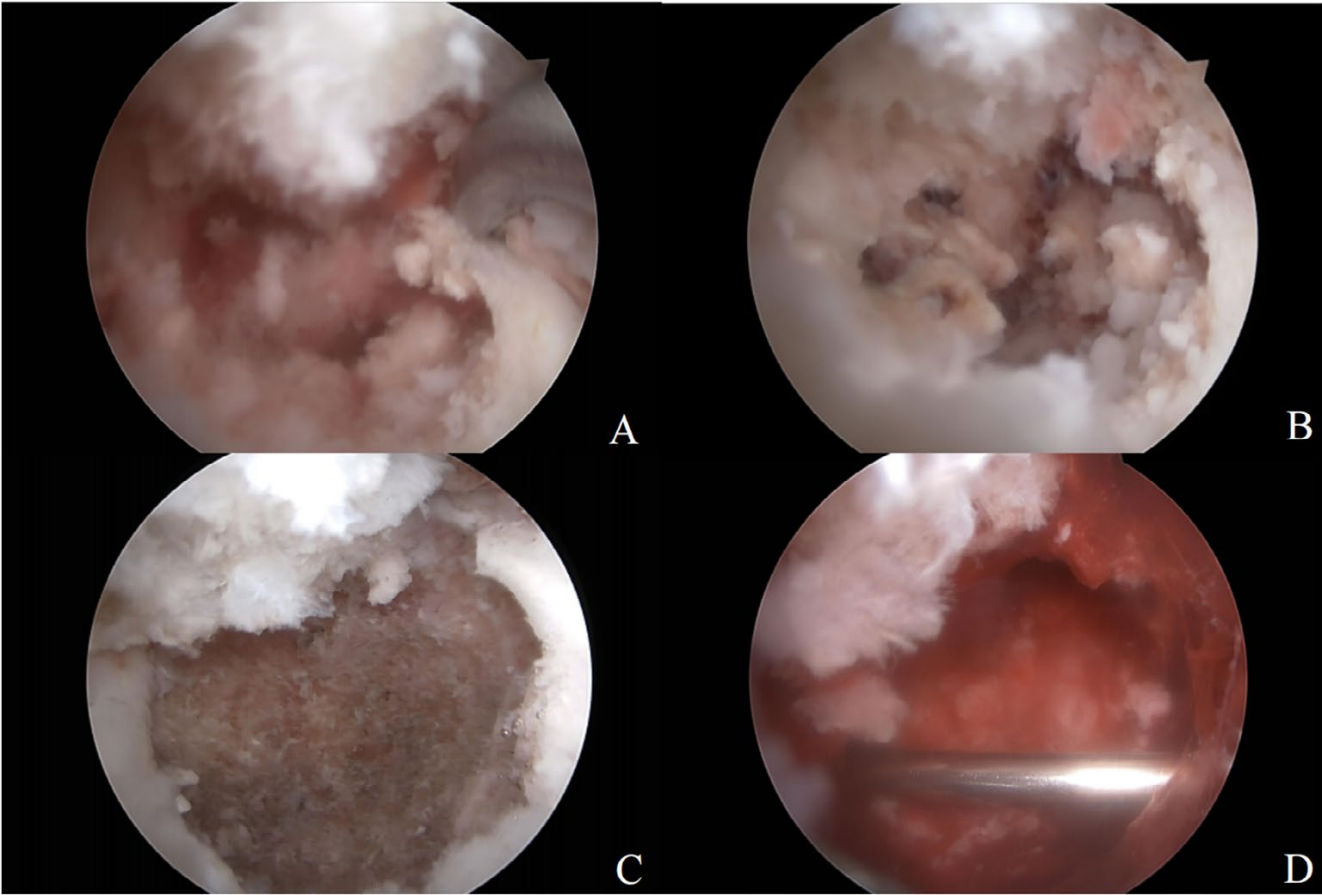
Arthroscopic procedures under direct vision: confirmed by probing the lesion area (A), thinned out cortex forming the roof of the lesion (B), complete debridement of tumor tissue is performed and healthy cancellous subchondral bone is reached (C) and graft bone was fitted into the defect and stabilized Kirschner wires (D).

Initially, a standard arthroscopic evaluation of the knee joint was conducted through the conventional anterolateral and anteromedial portals. The lesion was partly concealed by the anterior cruciate ligament and a vascular traction band is utilized to pull the anterior cruciate ligament and thereby expose the lesion (Figure 4A,B). The arthroscopic exploration revealed a hyperemic area located superior to the tibial intercondylar eminence, posterior to the cruciate ligament (Figure 4C) After synovium was resected, the location of the lesion was confirmed by probing the hyperemic area with a finger pin that pierced through the thinned out cortex forming the roof of the lesion (Figure 4D). After debriding the surrounding soft tissue, the lesion was de‐roofed using a shaver and curette (Figure 5A). Under direct vision, a lesion sharply separated from the adjacent bone and contains a mixture of soft, friable, gray material and hemorrhage was observed (Figure 5B). Small calcifications were observed on the surface of the lesion, presenting a granular and chalk‐like texture. Considering these morphological characteristics, a benign bone tumor was deemed as the most likely diagnosis. Tissue was obtained for histopathology with a grasper.

**FIGURE 6:**
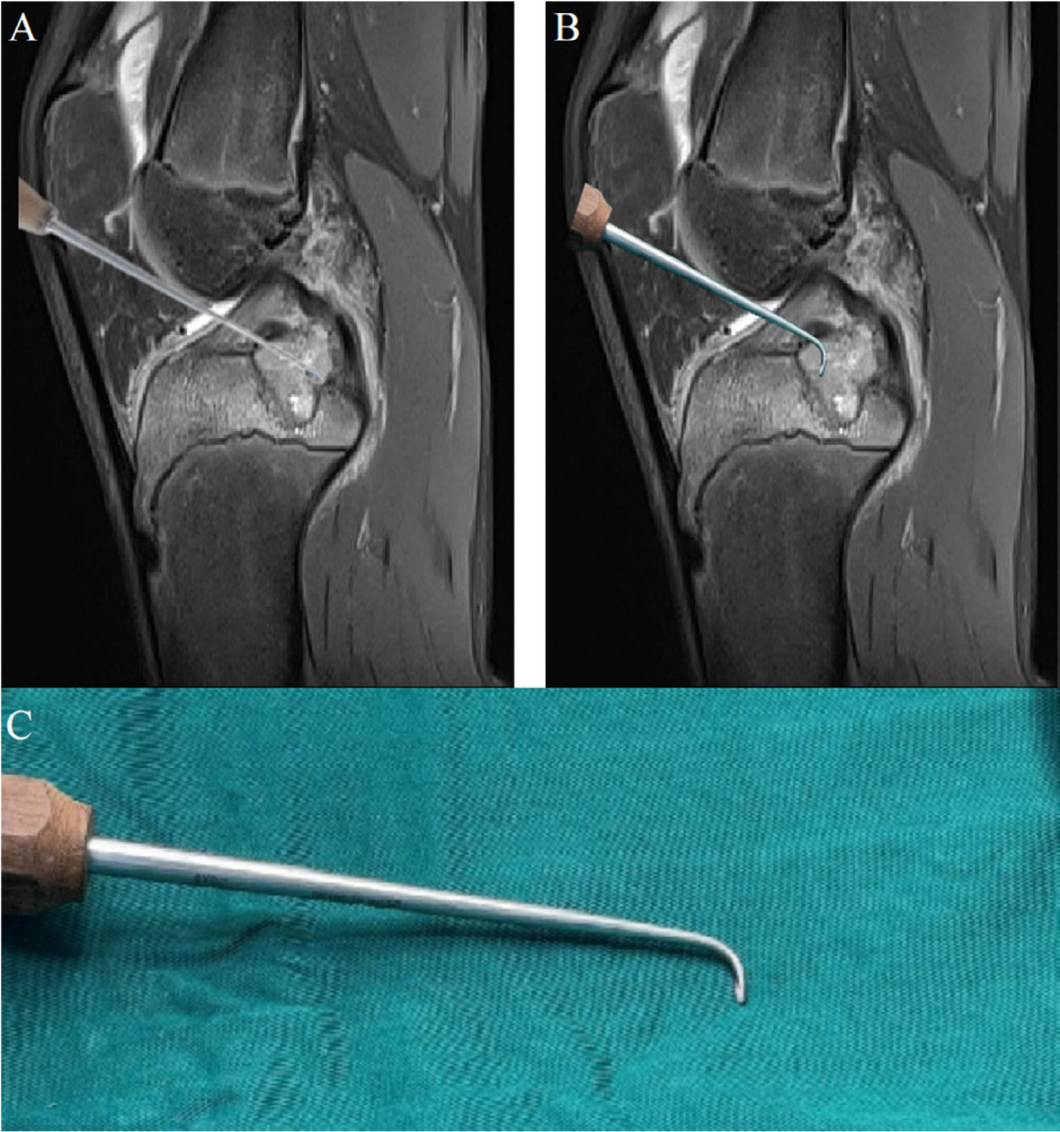
A shaver with a 90° bent angle was employed in surgery (C). A straight shaver cannot reach the anterior wall of the lesion (A). A shaver with a 90° bent angle can reach the anterior wall of the lesion (B).

The curettage of the anterior aspect of the lesion presents challenges due to its location; therefore, a shaver with a 90° bent angle was employed (Figure 6). The presence of residual tissue anterior to the lesion, however, is evident and a posterior medial approach was established. The remnants of the lesion were excised with a shaver under and the periphery was debrided under the arthroscopy until healthy cancellous bone became visible (Figure 5C). The graft bone came from the patient's iliac bone. The graft was prepared after removal of any adherent soft tissue and then reshape the graft into a cylindrical shape of approximately 2 cm. Finally, the graft bone was fitted into the defect and stabilized Kirschner wires and absorbable screw (Figure 5D). The incisions were closed, and the joint cavity was injected with ropivacaine, no drain was installed. Elastic bandage was applied. The surgery proceeded smoothly, and the excised tumor was sent for pathological biopsy. To prevent the dispersal of tumor cells into the joint, negative suction should be utilized during lesion curettage and continuous irrigation should be maintained throughout the entire surgical procedure.

**FIGURE A2:**
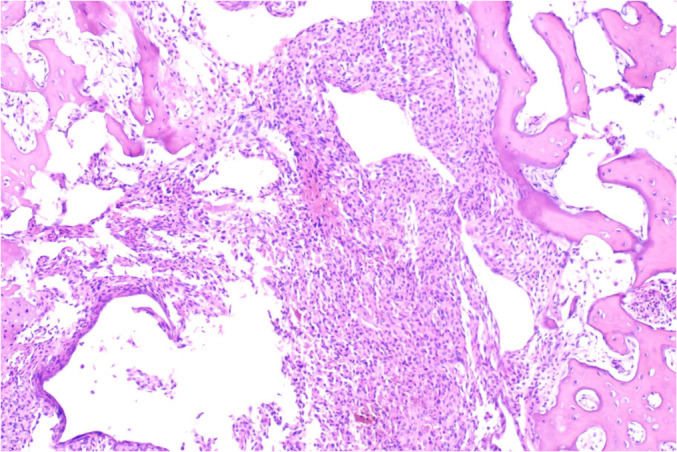
Histopathology showing cobblestone arrangement of chondrocytes on a chondroid matrix.

Histopathological examination revealed a cobblestone arrangement of chondrocytes on a chondroid matrix, exhibiting dystrophic calcification. These findings confirm the diagnosis of chondroblastoma (Figure A2).

**FIGURE A3:**
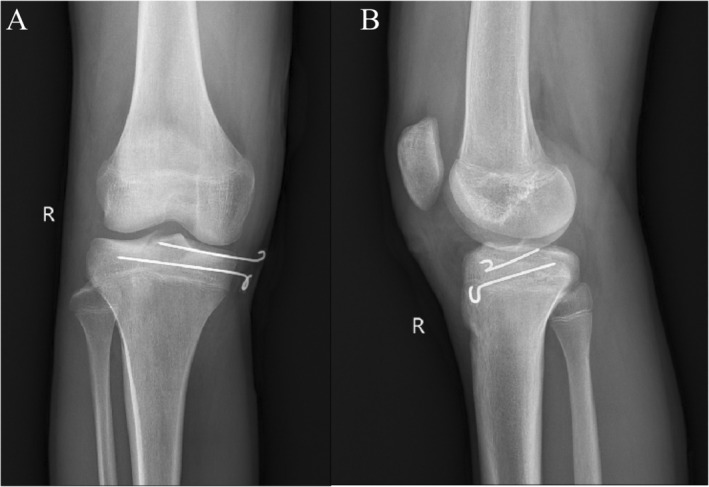
Radiographs of right knee 1 month after surgery: anteroposterior (A) and lateral (B) views.

**FIGURE A4:**
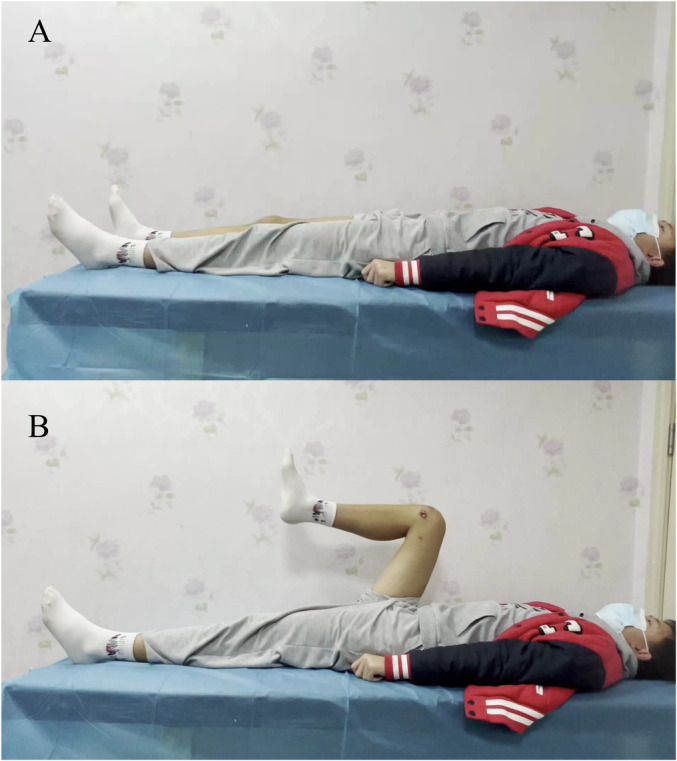
One month after surgery, the right knee can be extended 0° (A) and flexed 100° (B), with a Lysholm score of 67 for the right knee joint.

Postoperatively, partial weight‐bearing ambulation and knee range of motion exercises were started once the patient was comfortable in the first 2 weeks with the protection of a knee brace. He followed up regularly on an outpatient basis and did not show any evidence of an immunogenic reaction to the bone substitute, such as excessive drainage or joint effusion. During the one‐month postoperative follow‐up, the radiographs showed no evidence of recurrence (Figure A3) and the Lysholm score of the right knee was 67 (Figure A4). The Kirschner wires were removed upon observation of a good bone graft fusion substitute on follow‐up radiographs and CT images during the 2‐month postoperative assessment.

**FIGURE 7:**
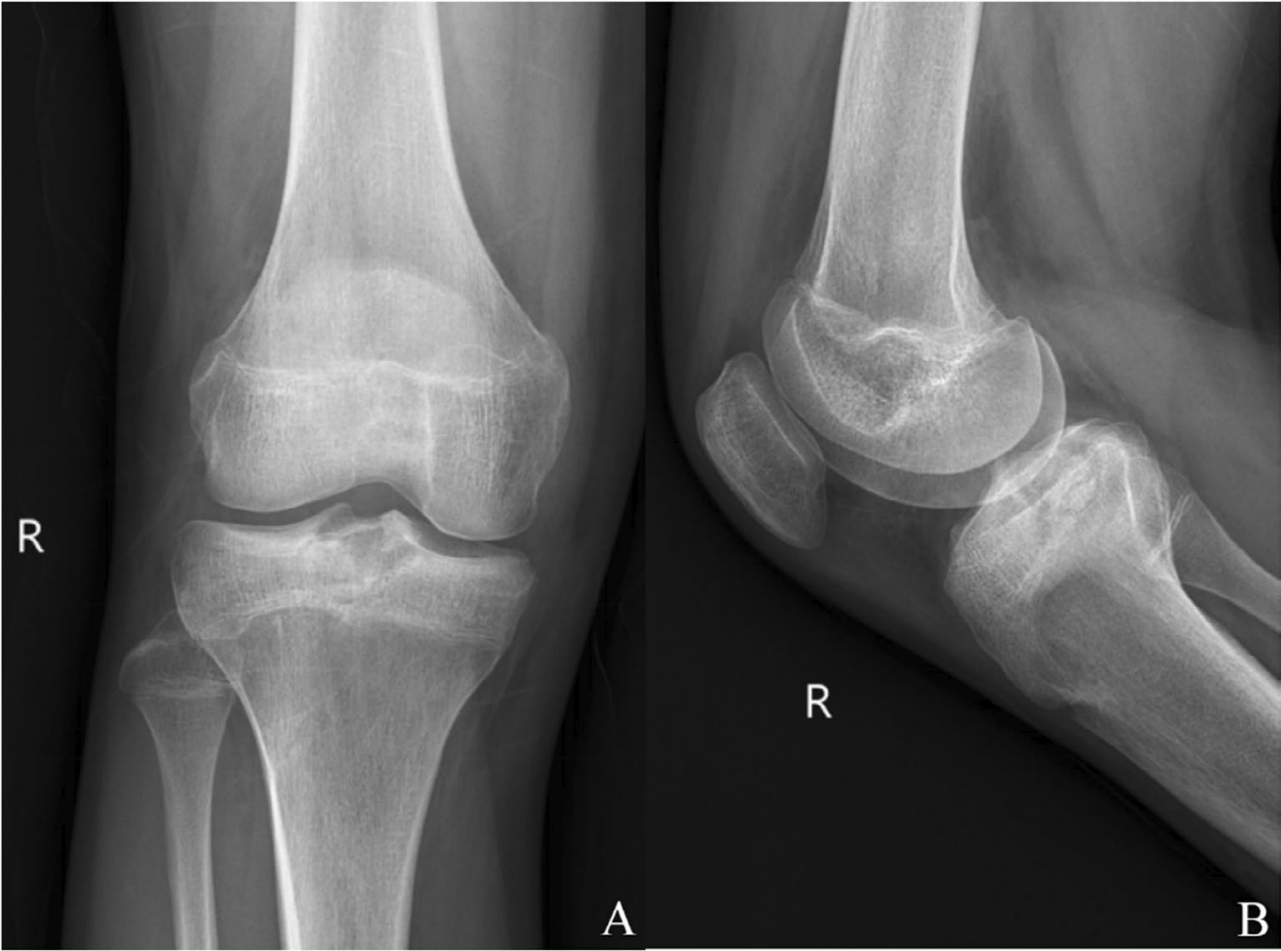
Radiographs of right knee showing a good osteointegration of the bone graft 3 months after surgery: anteroposterior (A) and lateral (B) views.

**FIGURE 8:**
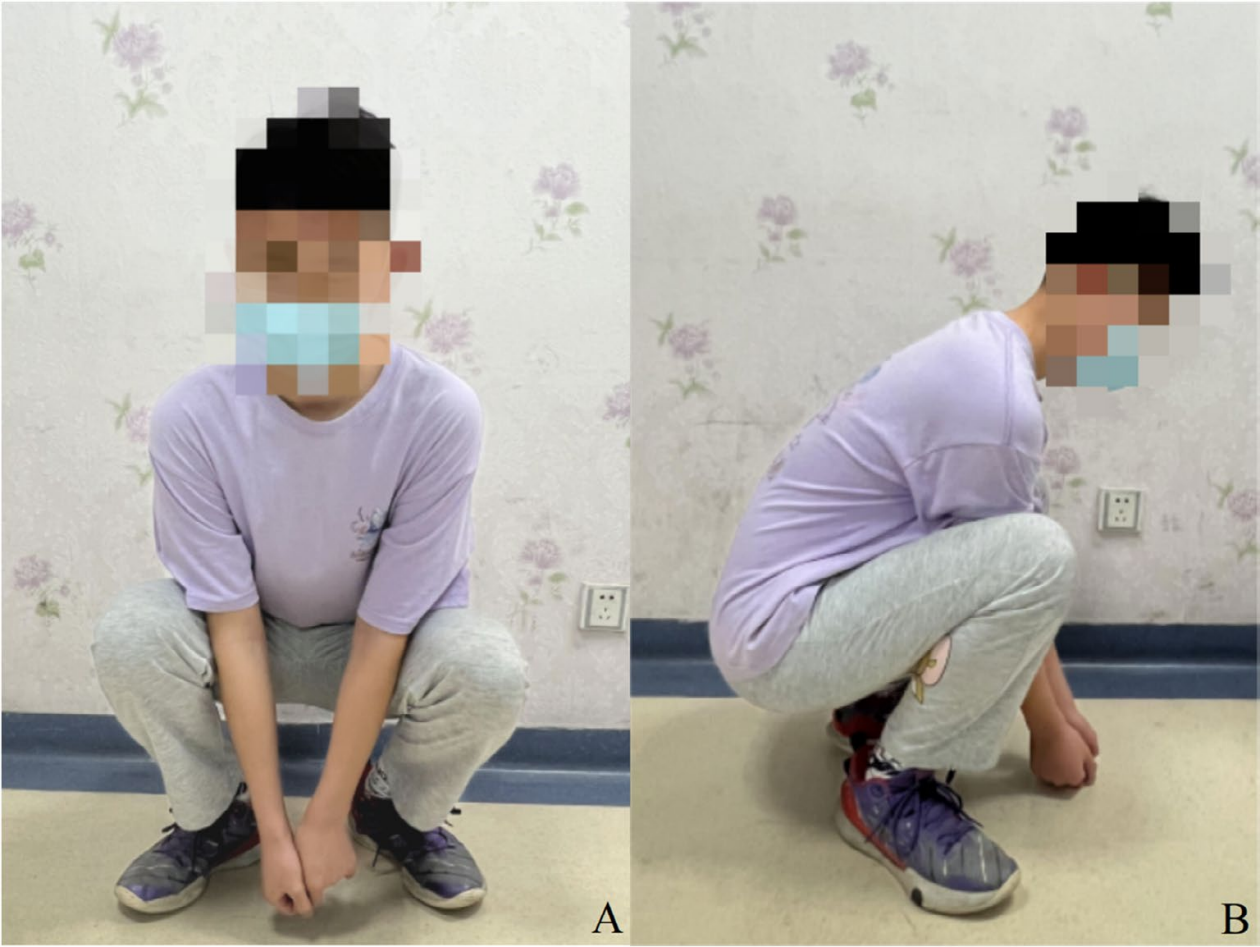
The 3‐months follow‐up after surgery demonstrated an excellent functionality of the knee joint: anterior (A) and lateral (B) view.

**FIGURE 9:**
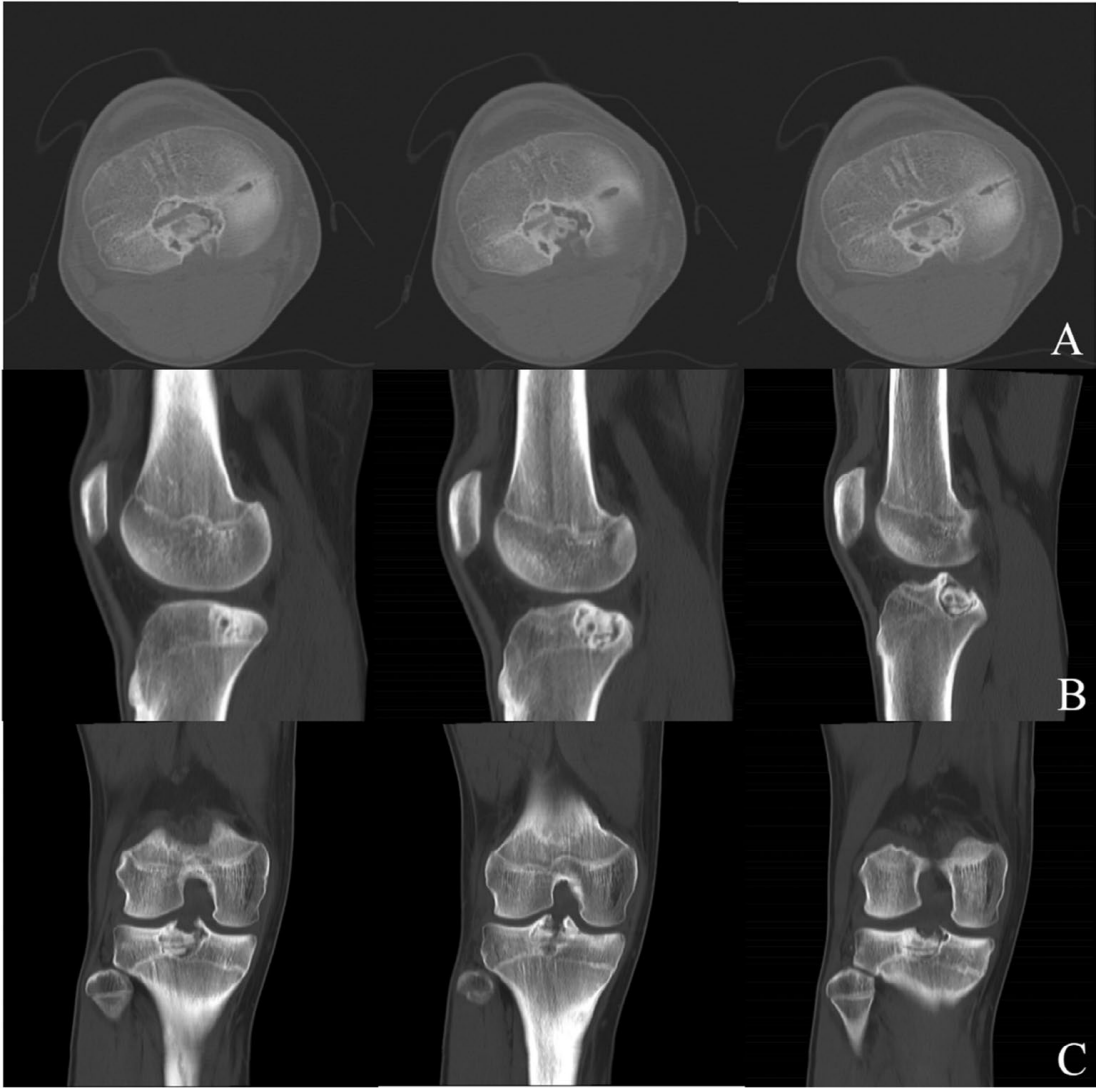
CT scan showing at 12 months of follow‐up of a good osteointegration of the bone graft: axial view (A), sagittal view (B), and coronal view (C).

**FIGURE 10:**
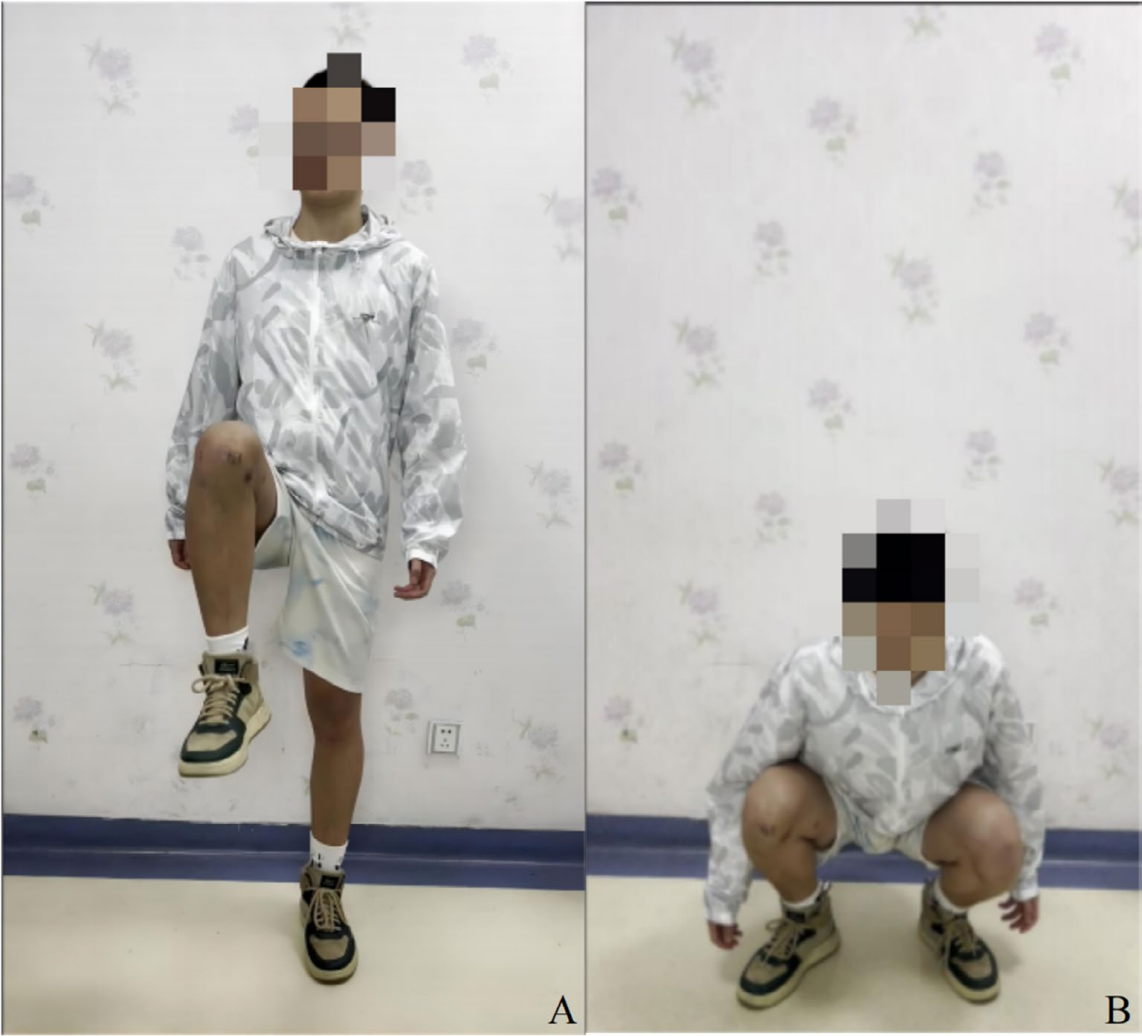
The 12‐months follow‐up after surgery demonstrated an excellent functionality of the right knee: flexed position of right knee (A) and Crouch of the patient (B).

Three months after surgery, the patient was able to walk while weight‐bearing, the radiographs showed a good bone fusion (Figure 7) and the Lysholm score of the right knee was 87 (Figure 8). At 12 months post‐surgery, CT scans revealed good fusion of the bone graft without any evidence of recurrence (Figure 9). The patient was asymptomatic with normal knee function. The right knee joint can flex up to 130°, returning to the normal level of flexibility. (Lysholm score = 100, Figure 10).

## 3 Discussion

Thechondroblastoma constitutes approximately 1% of primary bone tumors and exhibits a predilection for the epiphyses or apophyses of the long bones [1]. According to existing literature, the most prevalent sites for chondroblastoma occurrence are observed in proximal tibia or femur, distal femur, and proximal humerus [3, 10].

Chondroblastoma, although classified as a non‐malignant tumor, exhibits locally aggressive behavior [4] necessitating surgical intervention as an integral component of all treatment modalities. Meticulous curettage, either alone or in conjunction with chemical or mechanical adjuvants, along with bone‐grafting or cementation, represents the established standard of treatment [2]. The tumor is also known to have a relatively high local recurrence rate. Therefore, meticulous and assertive curettage is a crucial factor in achieving successful treatment outcomes because incomplete or insufficient curettage has been identified as the primary cause of recurrence [11]. The utilization of local adjuvants, such as liquid nitrogen [12], hydrogen peroxide [7], phenol [13], and cementing [14] (via its thermogenic effect), has been applied to reduce the recurrence rate. However, there is no evidence to support their use over aggressive curettage and grafting alone [15]. Furthermore, the application of phenol [16] may have deleterious effects on the integrity of articular cartilage in subchondral chondroblastoma and the thermogenic effects of cement present a substantial risk on the physis in skeletally immature patients [17]. Some scholars consider that recurrence is likely to be due to incomplete curettage; therefore, aggressive curettage should be the mainstay treatment rather than local adjuvant application after curettage [11]. One study reported a very low recurrence rate after the treatment of proximal humeral chondroblastoma. The authors emphasized the importance of meticulous and extensive curettage using curettes to remove all visible tumor tissue [12]. Although recurrence is one of the most common complications of chondroblastoma surgery, other types of complications should not be ignored. Neuropraxia was found after surgery which may related to stretching or compression of the nerve during surgery. Arthrosis was found after undergoing curettage and cementation for a tibial chondroblastoma. Neuropraxia was found after surgery, which may be related to the stretching or compression of the nerve during the procedure. Arthrosis was observed following curettage and cementation for a tibial chondroblastoma [18]. For children, premature closure of the growth plate due to tumor involvement is also a common complication [19]. Despite some scholars making improvements to surgical approaches, open surgery still presents similar complications [20]. However, smaller surgical incisions and approaches still result in improved postoperative function and recovery. Considering these complications, the use of arthroscopic techniques for minimally invasive surgery is a direction of development.

### 3.1 Review of Arthroscopic Treatment for Chondroblastoma

A few reports regarding the arthroscopic treatment of chondroblastoma have been published since 1992, when Cohen et al. initially reported excision of a proximal tibial chondroblastoma [9]. In the first arthroscopic treatment of chondroblastoma, the patient had a lesion in the right anterior tibial spine and a fixed flexion deformity of 30° in the knee, resulting in limited flexion to only 90°. Arthroscopic excision without bone graft of the chondroblastoma was performed. At review 1 year after operation, the knee was pain free with a full flexion and extension in right knee [9]. Then Stricker [21] applied percutaneous extra‐articular curettage with arthroscopy and bone grafting in three patients with femoral head chondroblastoma. This technique has evolved into a method known as “dry arthroscopy”. In the same year, another case report applied hip arthroscopy performed intra‐articular curettage and bone graft in femoral head [22] and medial femoral condyle chondroblastoma [23]. Based on the aforementioned studies, techniques for applying arthroscopic treatment in chondroblastoma can be classified into two different technical routes, arthroscopy and “dry arthroscopy”. In 2002, the percutaneous extra‐articular curettage with arthroscopy was successfully applied in a chondroblastoma in the calcaneus [24]. With the development of arthroscopy technique, arthroscopy was also applied in the treatment of ankle chondroblastoma. In 2010, Prado et al. [25] reported a case of patient with talar neck chondroblastoma, treated with arthroscopic resection. In addition to performing arthroscopic excision of chondroblastoma, the utilization of arthroscopic‐assisted biopsy and radiofrequency therapy is also implemented [26]. In last decade, arthroscopy assisted treatment of chondroblastoma has been demonstrated as an effective approach for the treatment of chondroblastoma, supported by several studies [27, 28, 29, 30, 31, 32]. The application of arthroscopic treatment in chondroblastoma is listed in Table 1. The above studies show that all patients successfully underwent arthroscopic surgery for chondroblastoma. Compared with the preoperative state, postoperative pain was effectively relieved and visual analog scale scores significantly improved.

**TABLE 1:** Application of arthroscopic treatment in chondroblastoma previously reported in the literature.

Study	Patient	Location	Intervention	Follow‐up time (months)	Recurrence	Complication
Cohen et al. [9]	1	Proximal tibia	Curettage without bone grafting	12	No	No
Thompson and Woodward [22]	1	Femoral head	Curettage without bone grafting	6	No	No
Stricker [21]	3	Femoral head	Curettage without bone grafting	4–38	No	No
Bal and Jones [23]	1	Medial femoral condyle	Curettage without bone grafting	60	No	No
Otsuka et al. [24]	1	Calcaneus	Curettage without bone grafting	24	No	No
Prado et al. [25]	1	Dorsal medial cortex of the talar neck	Curettage without bone grafting	3	No	No
Zoccali et al. [26]	1	Tibial spine area of the proximal tibia	Radiofrequency thermoablation	12	No	No
Miyazaki et al. [27]	1	Medial femoral condyle	Curettage with bone grafting	12	No	No
Errani et al. [28]	1	Lateral femoral condyle	Curettage and bone cement filling the defect	12	No	No
Choi et al. [31]	1	No mentioned in study	Curettage with bone grafting	19	No	No
Farouk et al. [32]	3	Distal femur	Hydrogen peroxide and bone grafts	26–45	No	No
Kellish et al. [29]	3	Distal femur	Curettage with bone graft	7–43	No	No
Acharya et al. [33]	1	Distal femur	Curettage and synthetic calcium hydroxyapatite granules	84	No	No
Chen et al. [30]	1	Talar	Curettage with bone grafting and platelet‐rich plasma‐fibrin glue	24	No	No
Morsy and Gawish [34]	No mentioned in study	Proximal Humeral	Curettage with bone grafting	No mentioned in study	No	No
Current study 2024	1	Tibial spine area of the proximal tibia	Curettage with bone grafting	12	No	No

### 3.2 Surgery Tips

While arthroscopy is an attractive option for treating lesions, the technique also has disadvantages, including the risk of intra‐articular tumor spread and poor biopsy specimen quality [23]. However, some studies have demonstrated that arthroscopy‐assisted resection of benign bone tumors may offer advantages such as reduced postoperative pain, improved cosmetic outcomes, early mobilization, and enhanced functional results [33, 34, 35].

The primary opponents to the arthroscopy assisted tumor surgery argue against the use of irrigation fluid. Because fluid flow may lead to a dissemination of tumor cells from the lesion into the joint. In order to mitigate this concern, a shaver under negative suction was applied during lesion curettage and continuous intraoperative irrigation during all procedures. The efficacy of this technique has been demonstrated in a similar case of knee chondroblastoma, lending support to its potential application in the present study [33]. Another technique almost eliminates this because “dry arthroscopy” do not use irrigation fluid [29]. Additionally, the decision to perform arthroscopy is heavily contingent upon tumor classification. As demonstrated in our case, the strategy using arthroscopy is to treat the cavity with an autologous bone graft substitute when the lesion is benign or transition to arthroscopy‐guided biopsy when an intraoperative diagnosis indicates a malignant lesion.

The second concertation of arthroscopy is the challenging bone grafting process for filling the tumor cavity with a reliable and compacted graft. In one study [34], the bazooka technique was employed, wherein an 8‐mm transparent cannula is utilized as a conduit to facilitate the transportation of bone grafts. This innovative approach enables precise loading of any desired quantity of bone graft chips into the tumor cavity, ensuring consistent impaction until complete nest filling is achieved. In our study, the lateral incision of the knee was extended by 2 cm to facilitate the insertion of the bone grift. Additionally, fixation is not usually required after bone grafting; however, in order to better graft bone fusion, we utilized Kirschner wire and absorbable screws for fixation in this study.

One study reported a 14‐year‐old girl with chondroblastoma involving the distal femoral epiphysis. An arthroscopic assisted lesion curettage was performed and a synthetic graft substitute was applied in the cavity management. The 7‐year follow‐up after the surgery demonstrated that the patient's symptoms have disappeared with a normal knee function, the bone graft area has fused well, and no recurrence evidences were overserved [33]. However, in our case, autologous iliac bone graft was used for better graft fusion. For the treatment of bone defects, bone graft is a common surgical procedure. Autologous bone is considered as the “perfect” bone‐grafting material because it combines all properties required in a bone‐graft material: osteo‐induction, osteogenesis and osteo‐conduction [36]. In addition, due to its being the patient's own tissue, autologous bone is histo‐compatible and non‐immunogenic, greatly reducing the possibility of immune reactions and transmission of infections. However, the collection requires additional surgical procedures and is associated with documented complications and discomfort for patients. There are also other disadvantages such as quantity limitations and high costs [37]. Moreover, chondroblastoma cases treated with curettage and synthetic bone substitutes exhibited a radiographic incidence of joint degeneration at a rate of 16.7% [38]. One recent report also suggests using low‐porosity *β*‐tricalcium phosphate as a reconstructive material after chondroblastoma resection surgery [39].

### 3.3 Prospect of Clinical Application and Limitations

For chondroblastoma occurring around the joints, the challenge of surgical treatment lies in its location and the surrounding soft tissues. For example, in this study, the lesion was located in the posterior area of the tibial eminence and was obstructed by cruciate ligaments and surrounding soft tissues. Performing open surgery may result in greater tissue damage and the formation of adhesions due to the need for a larger incision to adequately visualize the lesion. Arthroscopy demonstrated its technical feasibility and superiority in our case. For example, the improvement of the shaver with a 90° bent angle facilitates the debridement of the lesion. Another technique applied in this case involves the autogenous bone graft from the ilium and its fixation. Autogenous iliac bone grafting provides effective structural support, preventing subsequent collapse. The application of K‐wires and absorbable screws in fixation prevents the need for stage surgery. In addition, preoperative CT three‐dimensional reconstruction of the lesion helps us determine the size of autogenous bone grafts, avoiding damage caused by taking excessively large iliac bones. In our case, arthroscopy offers the benefit of observing the tumor directly with bright lighting in a field without blood. These experiences can help in the clinical application of arthroscopic treatment for chondroblastoma. At 4 weeks after arthroscopy, our patient was given the freedom of early partial weight bearing and early mobilization. The patient was able to engage in weight‐bearing activities earlier compared to open surgery. Significantly, a complete recovery of functionality has been observed within 8 to 12 weeks after arthroscopy.

In our study, we are delighted to report positive initial outcomes from the utilization of arthroscopic intervention for treating chondroblastoma in the tibial intercondylar eminence. Nevertheless, there exist limitations in this case that necessitate improvement. The primary limitation is that the arthroscopic surgery could result a dissemination of tumor cells into the joint. In order to avoid these occurrences, a negative suction and continuous intraoperative irrigation was employed during all procedures. The shaver, which was bent at a 90° angle, was modified during the surgery. However, careful consideration is necessary, as there is a possibility of breakage occurring during the procedure. The fixation methodology for graft bone can also be modified by utilizing a fixation technique for tibial eminence avulsion fractures [40]. Finally, the available evidence is inadequate to determine whether arthroscopic intervention for chondroblastoma reduced the rate of tumor recurrence. Hence, it is crucial for orthopedic surgeons to maintain a state of constant alertness. Further research studies with stronger evidence are necessary to substantiate this potential indication.

#### Conclusion

Based on the existing cases of arthroscopic treatment for chondroblastoma and the report of this case, arthroscopic treatment for chondroblastoma can be considered as a specific treatment option for certain patients. In some cases, this technique could be an effective alternative to open surgery.

## Author Contributions

C.X., S.Z., Z.G., L.W., Y.D. and J.L. designed research. C.X., S.Z., and Y.D. performed surgery and collected the data. C.X., S.Z., and L.W. wrote the manuscript. C.X., S.Z. and Z.G. reviewed and edited the manuscript. All authors read and approved the final document. C.X. and J.L. coordinated and supervised the production of the final document. All authors have read and agreed to the published version of the manuscript.

## Ethics Statement

This anonymous case report was published with the consent of the patient and his family.

## Consent

The authors have nothing to report.

## Conflicts of Interest

The authors declare no conflicts of interest.

## Supporting information


**Data S1:** Figures information.

## Data Availability

The datasets used and analyzed during the current study are available from the first or corresponding author upon reasonable request.
