# Supplementary figures and images for: Arthroscopic Management of Juxta‐Articular Proximal Tibial Chondroblastoma: A Case Report and Literature Review
Source: Orthop Surg. 2024 Nov 7;17(1):295–309. doi: 10.1111/os.14287 (PMC11735375; doi:10.1111/os.14287)

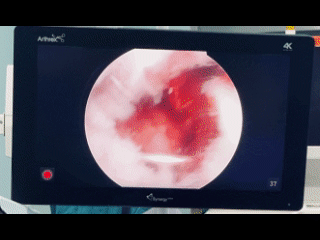

Supplement: Supplementary file 1 — Data S1: Figures information. [file OS-17-295-s001.zip › Check the condition of bone graft fixation.gif]

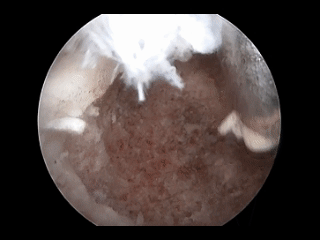

Supplement: Supplementary file 1 — Data S1: Figures information. [file OS-17-295-s001.zip › Complete debridement performed under arthroscopy.gif]

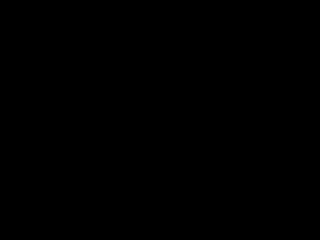

Supplement: Supplementary file 1 — Data S1: Figures information. [file OS-17-295-s001.zip › Curettage the anterior aspect of the lesion.gif]

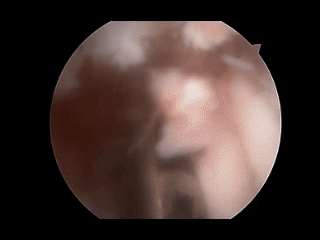

Supplement: Supplementary file 1 — Data S1: Figures information. [file OS-17-295-s001.zip › Curettage the lesion.gif]

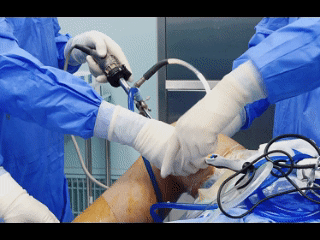

Supplement: Supplementary file 1 — Data S1: Figures information. [file OS-17-295-s001.zip › Debridement of the posterior aspect.gif]

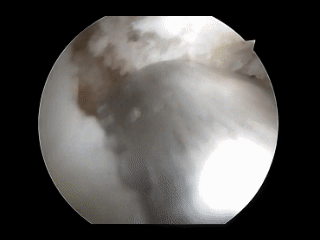

Supplement: Supplementary file 1 — Data S1: Figures information. [file OS-17-295-s001.zip › Exposure of the lesion 2(1).gif]

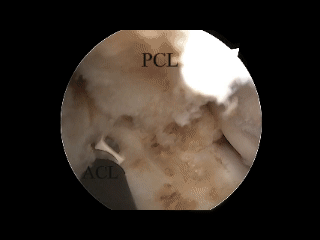

Supplement: Supplementary file 1 — Data S1: Figures information. [file OS-17-295-s001.zip › Exposure of the lesion.gif]

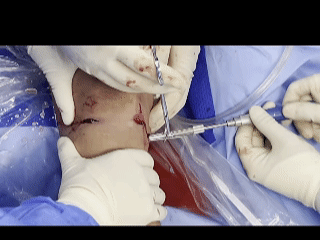

Supplement: Supplementary file 1 — Data S1: Figures information. [file OS-17-295-s001.zip › Install absorbable screw.gif]

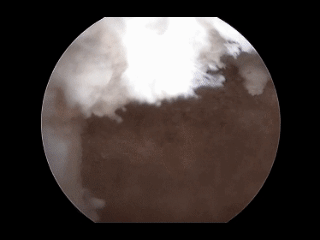

Supplement: Supplementary file 1 — Data S1: Figures information. [file OS-17-295-s001.zip › Install Kirschner pins for bone graft fixation.gif]

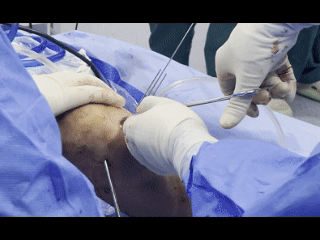

Supplement: Supplementary file 1 — Data S1: Figures information. [file OS-17-295-s001.zip › The graft bone was inserted into the defect 1.gif]

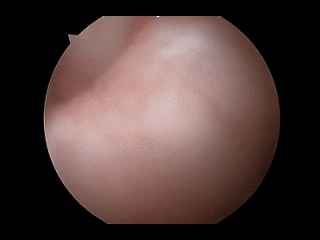

Supplement: Supplementary file 1 — Data S1: Figures information. [file OS-17-295-s001.zip › The graft bone was inserted into the defect 2.gif]
